# CCR5 Is Essential for NK Cell Trafficking and Host Survival following Toxoplasma gondii Infection

**DOI:** 10.1371/journal.ppat.0020049

**Published:** 2006-06-09

**Authors:** Imtiaz A Khan, Seddon Y Thomas, Magali M Moretto, Frederick S Lee, Sabina A Islam, Crescent Combe, Joseph D Schwartzman, Andrew D Luster

**Affiliations:** 1 Department of Microbiology, Immunology, and Parasitology, Louisiana State University Medical Center, New Orleans, Louisiana, United States of America; 2 Center for Immunology and Inflammatory Diseases, Division of Rheumatology, Allergy, and Immunology, Massachusetts General Hospital, Harvard Medical School, Boston, Massachusetts, United States of America; 3 Department of Pathology, Dartmouth Medical School, Lebanon, New Hampshire, United States of America; Stanford University, United States of America

## Abstract

The host response to intracellular pathogens requires the coordinated action of both the innate and acquired immune systems. Chemokines play a critical role in the trafficking of immune cells and transitioning an innate immune response into an acquired response. We analyzed the host response of mice deficient in the chemokine receptor CCR5 following infection with the intracellular protozoan parasite Toxoplasma gondii. We found that CCR5 controls recruitment of natural killer (NK) cells into infected tissues. Without this influx of NK cells, tissues from CCR5-deficient (CCR5^−/−^) mice were less able to generate an inflammatory response, had decreased chemokine and interferon γ production, and had higher parasite burden. As a result, CCR5^−/−^ mice were more susceptible to infection with T. gondii but were less susceptible to the immune-mediated tissue injury seen in certain inbred strains. Adoptive transfer of CCR5^+/+^ NK cells into CCR5^−/−^ mice restored their ability to survive lethal T. gondii infection and demonstrated that CCR5 is required for NK cell homing into infected liver and spleen. This study establishes CCR5 as a critical receptor guiding NK cell trafficking in host defense.

## Synopsis

*Toxoplasma gondii,* an obligate intracellular parasite, evokes a strong cellular immune response, which is critical for host protection. Chemokines and their receptors control the trafficking of immune cells to sites of infection. In this study, the authors examined the immune response to T. gondii in mice deficient in the chemokine receptor CCR5. CCR5-deficient mice exhibited a decreased inflammatory response, which led to a higher parasite burden and ultimate mortality. The deficiency in the immune response in CCR5-deficient mice was the result of decreased migration of natural killer (NK) cells into infected tissues. Transfer of normal NK cells into CCR5-deficient mice restored NK cell homing into infected tissue and the normal immune response capable of controlling T. gondii infection. These findings demonstrate an important role for CCR5 in trafficking the NK cells during acute T. gondii infection. Furthermore, since CCR5 is the main co-receptor for HIV-1 entry into cells, the results of this study raise a concern about the potential complication of impaired NK cell function in the clinical use of CCR5 antagonists, currently being developed as therapeutics for HIV-1 and inflammatory diseases. This may be of particular concern for HIV-1-infected patients coinfected with T. gondii or for those who acquire an acute T. gondii infection while taking a CCR5 antagonist.

## Introduction

A protective host response to intracellular pathogens, such as viruses and certain bacteria and protozoan parasites, requires a coordinated innate and acquired immune response. *Toxoplasma gondii,* an intracellular protozoan parasite of significant clinical importance, evokes a similar host response in mice and humans and therefore represents an excellent model system with which to use mouse genetics to dissect components of an effective immune response [1].

The innate immune response to T. gondii consists of neutrophil [2,3] and natural killer (NK) cell responses, while the acquired immune response requires interferon γ (IFNγ)-producing CD4 and CD8 T cells [4,5]. The critical role for NK cells in the innate response to T. gondii has been documented in interleukin (IL)-12 treated SCID mice [6] and in β2 microglobulin-deficient mice [7]. Early NK cell function is essential for host survival because it is an early source of IFNγ production [8].

To perform this essential role in innate host defenses, NK cells must traffic to the site of infection where they release IFNγ, which is critical for macrophage activation and increased expression of major histocompatibility complex class II [9,10]. NK cells are generated in the bone marrow, and after leaving this compartment are found in peripheral blood, spleen, and liver. They can, however, be induced to localize in tissues in response to infectious and inflammatory stimuli. The molecular mechanisms controlling NK cell trafficking are just beginning to be identified. Like other leukocytes, chemokines are thought to play an important role in NK cell trafficking. NK cells have been reported to express multiple chemokine receptors, including CXCR1–CXCR4, CCR4, CCR5, CCR7, CCR8 and CX_3_CR1; however, their individual roles in NK cell homing in vivo have not been delineated [11–14]. The chemokine CCL3/MIP-1α has been shown to be essential for early NK cell migration into the liver in response to mouse cytomegalovirus infection [15], although the NK cell receptor that mediates this important function of MIP-1α has not been elucidated. These NK cells are an important source of the IFNγ needed for the induction of chemokines, such as CXCL10/IP-10 and CXCL9/MIG, that control the trafficking of activated T cells into infected tissues [16]. Thus, chemokines are an important link between the innate and adaptive immune response operating at the level of NK cell recruitment into tissues and subsequent conversion of the NK cell response into a T cell response through the activity of IFNγ-inducible chemokines.

The chemokines and chemokine receptors responsible for NK cell trafficking in response to T. gondii infection have not been studied. However, as for the mouse cytomegalovirus model, we have found that IFNγ-inducible chemokines play a critical role in T cell trafficking into infected tissues in T. gondii infection [17]. We speculated that chemokines, and in particular CCL3/MIP-1α and its receptors CCR1 and CCR5, may act upstream of IFNγ, controlling the recruitment of NK cells into T. gondii-infected tissues. Recently, we evaluated CCR1-deficient mice and found a normal NK cell response in the tissues of the infected animals [3]. In the present study, we found that CCR5-deficient mice have an impaired ability to recruit NK cells into infected tissues, which leads to a diminished cytokine and chemokine response, higher tissue parasite burdens, and alterations in susceptibility to T. gondii infection dependent on genetic background.

## Results

### CCR5^−/−^ Mice in the C57BL/6 Background Survive Longer than Wild-Type Mice

**Figure 1:**
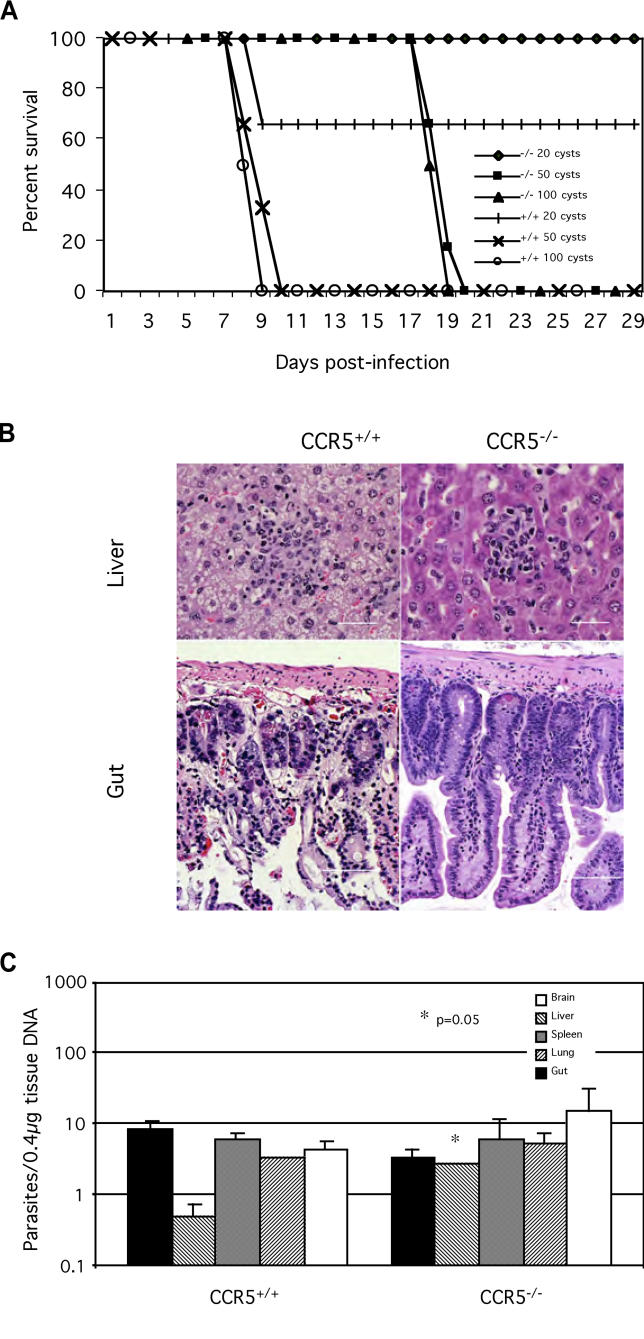
Survival, Histopathology, and Parasite Load of CCR5^−/−^ and CCR5^+/+^ Mice in a C57BL/6 Background following Infection with Different Doses of *T. gondii* (A) Survival. Female CCR5^−/−^ mice (5–7 wk old) and age-matched CCR5^+/+^ wild-type controls in the C57BL/6 background were challenged perorally with the cysts of the 76K strain of *T. gondii.* The animals were monitored for survival on a daily basis. There were six animals per group and the experiment was performed three times with similar results. (B) Histopathology. Photomicrographs of hematoxylin and eosin-stained liver and ileum of small bowel isolated from infected CCR5^−/−^ and CCR5^+/+^ mice. Liver of infected CCR5^+/+^ mouse reveals hepatocytes with extensive fatty degeneration. A nodule of inflammatory cells is seen in the center of the field, composed of lymphocytes and granulocytes. Hepatocyte morphology is preserved in liver of an infected CCR5^−/−^ mouse. A small nodule of inflammatory cells, largely composed of lymphocytes, is seen in the center of the field. Ileum of an infected CCR5^+/+^ mouse contains extensive epithelial necrosis and ulceration. Dilated blood vessels with hemorrhage are evident. The epithelial architecture is preserved in ileum of infected CCR5^−/−^ mouse. Bars: ileum, 100 μm; liver, 50 μm. (C) Level of parasite DNA*.* Groups of CCR5^−/−^ and CCR5^+/+^ mice on the C57BL/6 background were infected perorally with 20 cysts of *T. gondii*. At day 7 PI, small intestine, liver, spleen, lung, and brain from both CCR5^−/−^ and wild-type mice (three mice per group) were collected and the level of parasite load in the tissues was determined by competitive DNA PCR. The experiment was performed twice with similar results.

Previous studies by us and others have demonstrated that peroral infection of C57BL/6 mice with T. gondii cysts result in their death due to a cytokine-mediated hyperinflammatory response [18–20]. To determine the susceptibility of CCR5^−/−^ mice to T. gondii infection, CCR5^−/−^ and CCR5^+/+^ mice in the C57BL/6 background were infected perorally with 20, 50, and 100 cysts of *T. gondii.* All CCR5^+/+^ mice infected with 50 or 100 cysts died by day 8–10 postinfection (PI) (Figure 1A). In contrast, CCR5^−/−^ mice infected with the similar parasite doses did not die until day 18 PI (Figure 1A). When the infective dose was lowered to 20 cysts, about 30% of wild-type mice died (five of 18 mice from three separate experiments), while none of the CCR5^−/−^ mice succumbed to the infection at this dose. In addition, the number of T. gondii cysts in the brains of the survivors was enumerated after 1 mo PI, and CCR5^−/−^ mice had a 5-fold higher cyst number (2,563 ± 350) than control wild-type mice (538 ± 155) (*p* = 0.002) (unpublished data).

### CCR5^−/−^ Mice in the C57BL/6 Background Have a Reduced Hyperinflammatory Response

To determine whether the increased survival of CCR5^−/−^ mice in the C57BL/6 background was due to a diminished inflammatory response in these animals, tissues (liver and ileum of small bowel) from the CCR5^−/−^ and CCR5^+/+^ mice were histopathologically analyzed at day 7 PI. Inspection of multiple histological sections showed reproducible morphological changes. As expected, the liver of the wild-type mice had extensive fatty degeneration of hepatocytes, as has been previously described in the hyperimmune response in these mice [19]. Multiple mixed lymphocytic and granulocytic inflammatory nodules of 50–100 μm diameter were scattered throughout the parenchyma. Intracellular T. gondii were not observed, which is typical in this stage of the infection (Figure 1B). In contrast, CCR5^−/−^ mice showed normal hepatocyte morphology and small, scattered inflammatory nodules throughout the hepatic parenchyma, made up largely of lymphocytes (Figure 1B). Sections of the small bowel showed patchy, superficial necrosis and hemorrhage in the wild-type mice (Figure 1B), and normal bowel morphology in the CCR5^−/−^ mice (Figure 1B).

### Early Parasite Load Is Similar in CCR5^−/−^ and CCR5^+/+^ Mice on the C57BL/6 Background

T. gondii infection of the tissues of C57BL/6 mice has been reported to induce a hyperinflammatory response that leads to early death [19,20]. In the present studies we found that CCR5^−/−^ mice on this genetic background had a reduced T. gondii-mediated inflammatory reaction, which enabled them to survive the initial period. To further confirm that the death of the wild-type C57BL/6 mice was not due to increased parasite multiplication, tissues from both wild-type and CCR5^−/−^ C57BL/6 mice were analyzed for parasite load (Figure 1C). The assay was performed at day 7 PI, just before wild-type C57BL/6 mice began to die. With the exception of the liver, no difference in the parasite number was seen between the tissues of the C57BL/6 wild-type and CCR5^−/−^ mice, confirming the histopathological findings that the increase in mortality observed in CCR5^+/+^ mice was the result of the T. gondii-induced hyperimmune response, not of a difference in parasite burden. However, CCR5^−/−^ mice in the C57BL/6 challenged with 50 or 100 cysts ultimately succumbed to the infection at about day 18–19 PI. In contrast to the early hyperimmune death seen in wild-type C57BL/6 mice, CCR5^−/−^ C57BL/6 mice apparently died from an increased parasite burden, as these mice exhibited a 10- to 100-fold increase in tissue (liver, lung, and spleen) parasite load at day 17 PI compared to day 7 PI. Moreover, histopathological analysis of these mice at this time point indicated high parasite multiplication with intracellular parasites readily identified in the liver (unpublished data).

### CCR5^−/−^ Mice on a Mixed C57BL/6x129 Background Are Susceptible to T. gondii Infection

**Figure 2:**
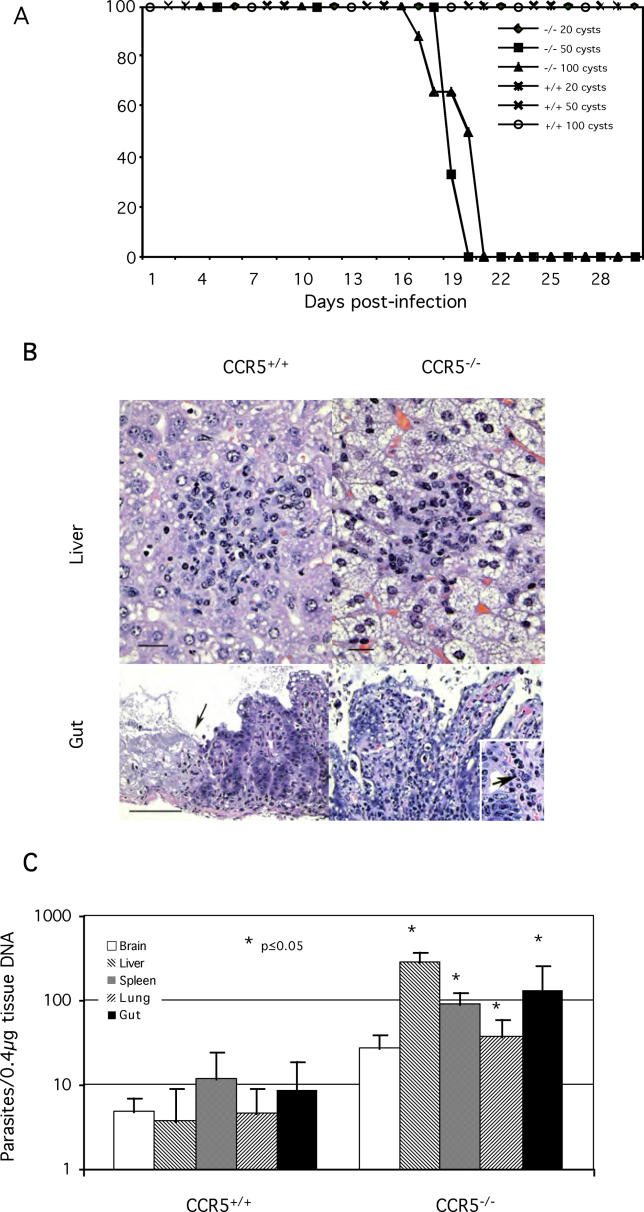
Survival, Histopathology, and Parasite Load of CCR5^−/−^ and CCR5^+/+^ Mice in a C57BL/6x129 Background following Infection with Different Doses of *T. gondii* (A) Survival. Female CCR5^−/−^ mice (5–8 wk old) and age-matched CCR5^−/−^ wild-type littermate controls, both in a C57BL/6x129 background, were challenged perorally with the cysts of the 76K strain of *T. gondii*. The animals were monitored for survival on daily basis. There were six animals per group and the experiment was performed three times with similar results. All CCR5^+/+^ mice survived at the three doses of cysts tested. (B) Histopathology. Photomicrographs of hematoxylin and eosin-stained liver and ileum of small intestine isolated from infected CCR5^+/+^ mice and CCR5^−/−^ mice at day 14 PI. Wild-type liver: Moderate fatty change is seen in hepatocytes, with small foci of mixed polymorphonuclear and mononuclear infiltration. Bar, 10 μm. CCR5^−/−^ liver: Severe fatty changes are seen in hepatocytes with mononuclear and polymorphonuclear inflammatory nodules. Bar, 10 μm. Wild-type small intestine: The superficial mucosa is missing in some places (arrow) and no parasite multiplication is evident. Bar, 100 μm. CCR5^−/−^ small intestine. The superficial mucosa is largely missing and a mixed inflammatory infiltrate including polymorphonuclear cells is evident within the lamina propria and superficial mucosa. Bar, 100 μm. Inset: Higher magnification with arrow pointing to tachyzoites in lamina propria of CCR5^−/−^ mice. Bar, 75 μm. (C) Level of parasite DNA*.* Groups of CCR5^−/−^ mice and wild-type littermate controls both on a C57BL/6x129 background were infected perorally with 20 cysts of *T. gondii*. At day 14 PI, small intestine, liver, spleen, lung, and brain from both CCR5^−/−^ and wild-type mice (three mice per group) were collected and the parasite load in the tissues was determined by competitive PCR. The experiment was performed twice with similar results.

To further establish that CCR5^−/−^ mice have an impaired host response to *T. gondii,* we studied the response of CCR5^−/−^ mice in the C57BL/6x129 background. Based on our prior experience, mice on this genetic background do not die from T. gondii infection, even at an infective dose of over 100 cysts of the 76 K strain. Mice on this mixed genetic background also do not exhibit a parasite-induced hyperimmune response. CCR5^−/−^ mice on the C57BL/6x129 background and wild-type control littermates were challenged with different doses of T. gondii cysts as described above. As expected, none of the wild-type mice succumbed to infection following infection with 20, 50, or even 100 cysts (Figure 2A). In contrast, all of the CCR5^−/−^ mice challenged with either 50 or 100 cysts died between days 17 and 21 PI (Figure 2A). None of the wild-type or CCR5^−/−^ mice died when challenged with a dose of 20 cysts.

### CCR5^−/−^ Mice Have a Higher Parasite Burden in the Tissues

Liver and ileum of small bowel from CCR5^−/−^ and wild-type littermate control mice were histopathologically analyzed at day 14 PI. Tissues from CCR5^−/−^ mice demonstrated severe injury, suggestive of uncontrolled parasite replication (Figure 2B). The intestinal lesions of CCR5^−/−^ mice were also characterized by a polymorphonuclear granulocyte-rich inflammatory infiltrate, demonstrating continued acute infection, which differs from the infiltrate seen in the littermate wild-type controls (Figure 2B). The lesions in the ileum of CCR5^−/−^ mice indicated high parasite multiplication, and intracellular parasites were readily identified (Figure 2B). Conversely, both tissues from wild-type mice exhibited only moderate inflammatory reaction, typical of mice that are recovering from acute infection.

To further confirm the observation that the absence of CCR5 compromised the ability of mice to inhibit parasite multiplication, tissues from CCR5^−/−^ and CCR5^+/+^ mice on a C57BL/6 x129 background were assayed for parasite burden at days 7 and 15 PI. Interestingly, unlike C57BL/6 CCR5^−/−^ mice (Figure 1C), CCR5^−/−^ mice in the C57BL/6 x129 at day 7 exhibited an increased parasite load (~10-fold more parasite DNA) in the liver, lung, and small intestine (unpublished data). The parasite load in CCR5^−/−^ mice, in comparison to wild-type littermate mice, was further elevated at day 15 PI in all tissues examined (Figure 2C), demonstrating that CCR5 was required for effective parasite control.

### Impaired NK Cell Response in CCR5^−/−^ Mice

**Figure 3:**
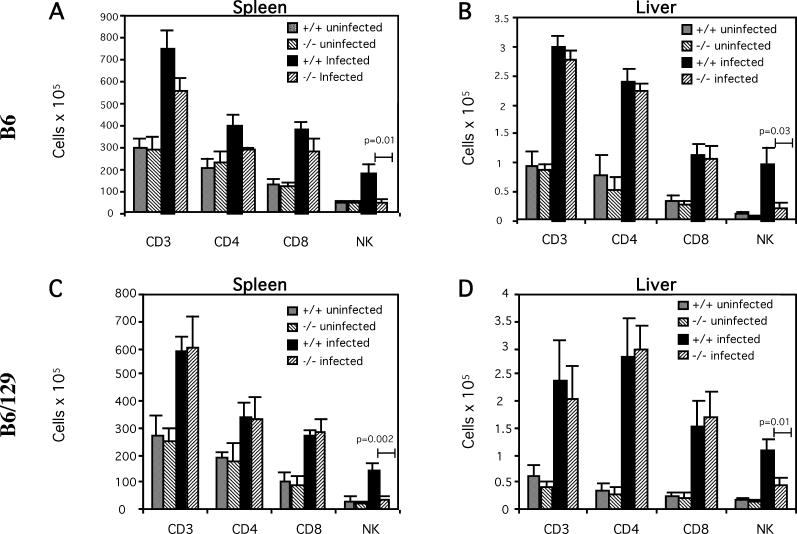
T Cell and NK Cell Numbers in the Spleen and Liver of T. gondii-Infected CCR5^−/−^ and CCR5^+/+^ Mice in the C57BL/6 and C57BL/6x129 Backgrounds Cell numbers. T cell and NK cell numbers in spleen (A and C) and liver (B and D) of mice infected with 15 cysts and harvested 7 d PI. Single-cell suspensions of spleen and hepatic lymphocytes were prepared and phenotyped for the expression of CD3, CD4, CD8, and NK1.1 by FACS analysis. The experiment was performed twice with similar results (three or four mice per group)*.*

**Figure 4:**
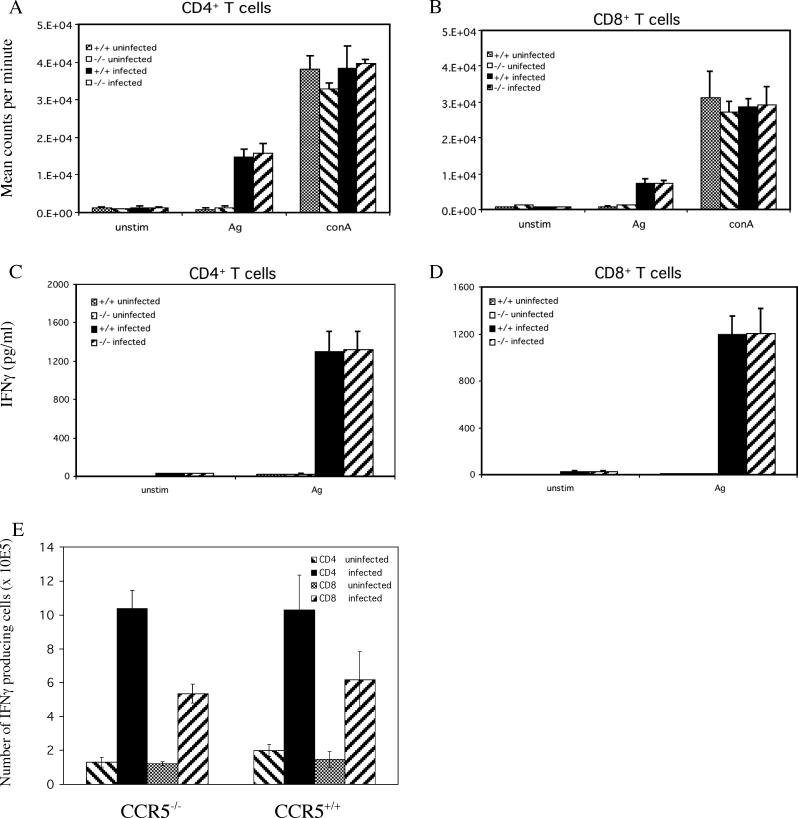
CD4^+^ and CD8^+^ T Cell T. gondii-Induced Proliferation and IFNγ Production (A and B) Proliferation. Magnetically purified positively selected CD4^+^ (A) and CD8^+^ (B) T cells (>95% pure) isolated from the spleens of C57BL/6x129 on day 7 PI. Cells were stimulated either with ConA (2.5 μg/ml) or *Toxoplasma* lysate antigen (15 μg/ml). After 72 h incubation, proliferation was measured by ^3^H thymidine incorporation. Data are represented as mean cpm ± standard deviation and are representative of two experiments. (C and D) IFNγ secretion. 10^6^ purified CD4^+^ (C) and CD8^+^ (D) T cells from 7 d infected C57BL/6x129 mice were cultured in presence of 15 μg/ml of *Toxoplasma* lysate antigen and irradiated feeder cells (5 × 10^5^ cells/well) in 24-well plates. After 72 h of incubation the supernatants were collected, centrifuged, and assayed for IFNγ production by ELISA. (E) Intracellular IFNγ production. Female CCR5^−/−^ (5–8 wk old) and wild-type mice were infected perorally with T. gondii cysts and splenocytes were harvested at day 7 PI, pooled (three mice per group), and cultured in vitro with phorbol 12-myristate 13-acetate, ionomycin, and monensin for 4 h. The cultured cells were stained for CD4 or CD8 before intracellular staining for IFNγ. Data are presented as percentage (mean ± standard deviation) of CD4^+^ or CD8^+^ T cells positive for IFNγ and are pooled from two experiments.

We sought to determine whether the reduced inflammatory response in the CCR5^−/−^ mice was due to the inability of the immune cells to traffic into infected sites. Spleens and livers of CCR5^−/−^ and CCR5^+/+^ mice on the C57BL/6 background were isolated at day 7 PI. Single-cell preparations of splenocytes and hepatic lymphocytes were stained with labeled antibodies against leukocyte markers and analyzed by FACS. Wild-type mice on both C57BL/6 and C57BL/6x129 backgrounds showed the expected influx in total T cell (CD3^+^) and CD4^+^ and CD8^+^ T cell subset populations in response to T. gondii infection in the spleen (Figure 3A and 3C) and liver (Figure 3B and 3D). CCR5^−/−^ mice in both the C57BL/6 and C57BL/6 x129 backgrounds also had a comparable increase in CD3^+^, CD4^+^, and CD8^+^ T cells in infected tissue. To determine whether lack of CCR5 had an effect on the generation of antigen-specific immune responses against *T. gondii,* antigen-specific proliferative responses were measured using fractionated CD4^+^ and CD8^+^ splenocytes from C57BL/6 x129 wild-type and CCR5^−/−^ mice. No significant differences in antigen-specific proliferation of CD4^+^ or CD8^+^ T cells in the two strains of mice were observed (Figure 4A and 4B). In a separate study, supernatants from CD4^+^ and CD8^+^ T cell cultures were collected 72 h after antigenic stimulation, and levels of IFNγ determined by ELISA. Similar to proliferation studies, CD4^+^ and CD8^+^ T cells from both CCR5^−/−^ and wild-type mice secreted equivalent amounts of IFNγ in response to antigenic stimulation (Figure 4C and 4D). Furthermore, wild-type and CCR5^−/−^ had a comparable increase in the number of IFNγ-producing CD4^+^ and CD8^+^ T cells found in the spleen 7 d after T. gondii infection as determined by intracytoplasmic cytokine staining (Figure 4E). These data indicate that CCR5 deficiency did not have a significant impact on the generation of an antigen-specific T cell response against the parasite.

Although no differences in T cell immunity was observed between wild-type and CCR5^−/−^ mice, a deficient NK cell response to T. gondii was apparent in the CCR5^−/−^ mice. Infected wild-type C57BL/6 and C57BL/6x129 mice exhibited a 3.5- to 8-fold increase in the NK cell population in the spleen and liver, respectively, as compared to uninfected animals on day 7 PI. In contrast, however, CCR5^−/−^ mice on both the C57BL/6 and C57BL/6x129 backgrounds did not have this increase in NK cell numbers in the spleen (Figure 3A and 3C) and liver (Figure 3B and 3D) in response to T. gondii infection. These data reveal a critical role for CCR5 in NK cell trafficking into the liver and spleen in vivo.

**Figure 5:**
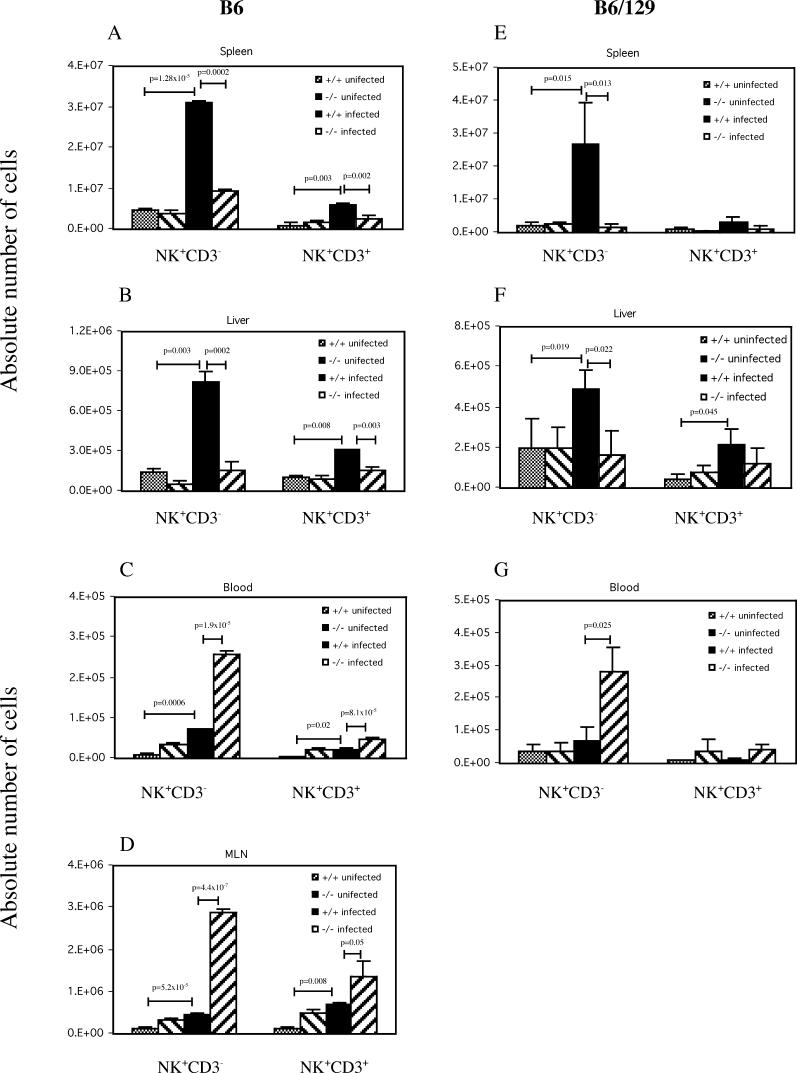
NK and NK T Cell Numbers in T. gondii-Infected CCR5^−/−^ and CCR5^+/+^ Mice in the C57BL/6 and C57BL/6x129 Backgrounds CCR5^+/+^ and CCR5^−/−^ mice in C57BL/6 and C57BL/6 × 129 backgrounds (three mice per group) were infected with 15 T. gondii cysts. At day 7 PI, lymphocytes from spleen (A and E), liver (B and F), blood (C and G), and mesenteric lymph nodes (D) were isolated and phenotyped for CD3^+^ and NK1.1^+^ by FACS analysis. NK1.1^+^CD3^−^ and NK1.1^+^CD3^+^ cell numbers are graphed. The experiment was performed twice with similar results.

Since the NK1.1 marker is also found on NK T cells in addition to true NK cells, we determined whether the increase in the NK cell population seen during T. gondii infection was restricted to classical NK cells (CD3^−^) or included NK T cells (CD3^+^). Spleen, liver, lymph nodes, and blood from infected wild-type and CCR5^−/−^ mice were analyzed for NK1.1^+^CD3^+^ and NK1.1^+^CD3^−^ cells on day 7 PI. In agreement with our above-described findings, we detected a 4- to 7-fold increase in NK1.1^+^CD3^−^ cells in the spleen and liver of wild-type mice of both strains at day 7 following infection with T. gondii (Figure 5A, 5B, 5E, and 5F). In contrast, infected CCR5^−/−^ mice had essentially no increase in numbers of NK1.1^+^CD3^−^ cells in the spleen and liver. Interestingly, however, infected CCR5^−/−^ mice in both strains had 4-fold more NK1.1^+^CD3^−^ cells in the blood (Figure 5C, *p* = 1.9 × 10^−5^; Figure 5G, *p* = 0.025) and mesenteric lymph nodes compared to infected wild-type mice (Figure 5D, *p* < 4.4 × 10^−7^). These observations suggest that CCR5^−/−^ mice generated and mobilized an NK cell response to *T. gondii,* but were unable to recruit these cells into tissue sites of infection. There was also a statistically significant 3- to 5-fold increase in NK1.1^+^CD3^+^ cells in the spleen, liver, and lymph nodes following infection in mice with C57BL/6 background. Similar to what was observed for NK1.1^+^CD3^−^ cells in CCR5^−/−^ mice, NK1.1^+^CD3^+^ cells were found in higher numbers in the blood and mesenteric lymph nodes and in lower numbers in the spleen and liver. However, in C57BL/6x129 mice, a significant increase in the NK1.1^+^CD3^+^ cells in response to T. gondii infection was observed only in the liver of wild-type mice (Figure 5F). These observations suggest that CCR5^−/−^ mice generated and mobilized an NK T cell response to *T. gondii,* but were unable to recruit these cells into tissue sites of infection.

### Early Burst of CCR5 Ligands in Infected Tissue

**Figure 6:**
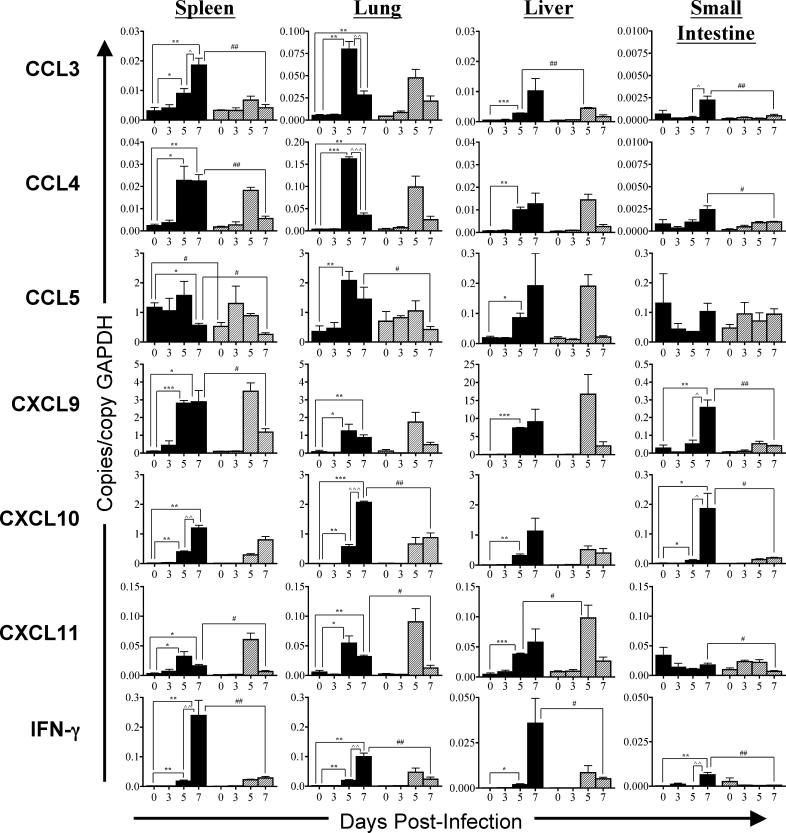
Expression of CCR5 and CXCR3 Chemokine Ligands and IFNγ in *T. gondii*-Infected Mice Quantitative PCR was performed on total RNA isolated from spleen, lung, liver, and small intestine of wild-type control and CCR5^−/−^ C57BL/6 mice (female, 5–6 wk old, three or four mice per group) infected orally with 15–50 cysts of 76K strain of *T. gondii*. Tissues were harvested from uninfected and at days 3, 5, and 7 PI. Data are displayed as copies of cytokine or chemokine per copies of GAPDH, with wild type in black bars and CCR5^−/−^ in hatched bars ± standard error of the mean. Statistics were performed using Student's t-test. Sets of symbols: * indicates comparisons with uninfected wild type, ^ indicates comparisons between days 5 and 7 PI; and # indicates comparisons between wild type and CCR5^−/−^. * *p* < 0.05, ** *p* < 0.01, *** *p* < 0.001, ^ *p* < 0.05, ^ ^ *p* < 0.01, ^ ^ ^ *p* < 0.001, # *p* < 0.05, ## *p* < 0.01, ### *p* < 0.001.

We examined the expression of the three known CCR5 ligands, MIP-1α/CCL3, MIP-1β/CCL4, and RANTES/CCL5, in infected wild-type and CCR5^−/−^ C57BL/6 mice on days 3, 5, and 7 post T. gondii infection. Mice were sacrificed at days 0, 3, 5 and 7 PI and tissues (spleen, lung, liver, and small intestine) were assayed for chemokine mRNA by quantitative PCR (QPCR) (Figure 6). We detected a similar increase in CCL3 and CCL4 mRNA levels in the spleen, lung, and liver on day 5 PI in both wild-type and CCR5^−/−^ mice. CCL5 was similarly increased in lung and liver on day 5 in both wild-type and CCR5^−/−^ mice. Interestingly, levels of CCL3 continued to rise on day 7 in spleen and liver of wild-type, but not of CCR5^−/−^ mice, leading to differences in the levels of CCL3 on day 7 in the spleen and liver, at which time we found differences in NK cell numbers in those tissues. CCL5 also had a similar increase in infected wild-type and CCR5^−/−^ mice in the lung and liver on day 5. However, by day 7 the levels in CCR5^−/−^ mice decreased, while the levels in the wild-type did not. Thus, we found that an early burst of CCR5 ligand production occurred in both wild-type and CCR5^−/−^ mice following infection that likely accounted for the recruitment of CCR5^+^ NK cells into these tissues by day 7. However, without this influx of NK cells in the CCR5^−/−^ mice, the expression of these ligands decreased by day 7, suggesting that a positive feedback loop exists between NK cells and the production of CCR5 ligands that lead to NK cell recruitment. It is also worth noting that we detected very low levels of CCL3 and CCL4 in the small intestine, which might explain our inability to detect NK cell recruitment into this tissue following T. gondii infection. CCL3 and CCL4 are induced in many cell types in response to proinflammatory stimuli, such as lipopolysaccharide and tumor necrosis factor α. Decreased levels of CCL3 and CCL4 in infected CCR5^−/−^ mice at day 7 thus likely reflects a diminished inflammatory response in CCR5^−/−^ mice. CCL5 is also induced by multiple inflammatory stimuli and is also constitutively expressed in T cells. Thus, decreased CCL5 levels in CCR5^−/−^ mice on day 7 likely also reflects a decreased inflammatory response in the CCR5^−/−^ mice.

### Diminished IFNγ and IFNγ-Inducible CXCR3 Chemokine Ligands in CCR5^−/−^ Mice at Day 7 PI

As NK cells are an important source of IFNγ during early T. gondii infection [8], we analyzed the mRNA expression of IFNγ, and the IFNγ-inducible CXCR3 chemokine ligands IP-10/CXCL10, MIG/CXCL9 and I-TAC/CXCL11 in wild-type and CCR5^−/−^ mice. Mice were sacrificed at day 0, 3, 5 and 7 PI and tissues (spleen, lung, liver, and small intestine) were assayed for chemokine mRNA by QPCR (Figure 6). As others and we have described previously, IFNγ, CXCL10, and CXCL9 were significantly induced in all organs tested following infection. CXCL11 was also induced in the spleen, lung, and liver. In all cases, induction of these cytokines was similar in wild-type and CCR5^−/−^ mice up to day 5. However, as was seen above for the CCR5 ligands, by day 7 the levels of IFNγ and IFNγ-inducible chemokines markedly diminished in organs harvested from infected CCR5^−/−^ mice. Thus, a reduction of NK cell infiltration into tissues resulted in decreased IFNγ production in response to T. gondii infection in the CCR5^−/−^ mice, which led to down-regulation of IFNγ-inducible genes, such as those encoding CXCL9, CXCL10, and CXCL11.

### CCR5 Acts Downstream of IL-12

**Figure 7:**
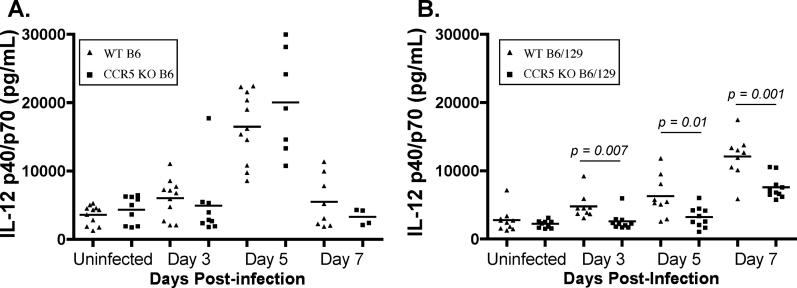
Serum IL-12 Levels for Wild-Type and CCR5^−/−^ Mice Wild-type (WT) and CCR5^−/−^ (CCR5 KO) mice in the C57BL/6 background (A) and C57BL/6x129 background (B) are represented. Serum IL-12 levels were measured by ELISA before infection or at days 3, 5, and 9 PI with 50 cysts of strain 76K *T. gondii.* Each data point was measured in duplicate and indicates IL-12 levels for an individual mouse. The bar represents the average at each time point. Statistics were performed using Student's t-test.

CCR5^−/−^ mice are known to have a diminished IL-12 response following T. gondii infection [21]. As IL-12 is important for NK cell function [22], a decrease in NK cell function in T. gondii-infected CCR5^−/−^ mice could be the result of suboptimal IL-12 production in these animals. To investigate this possibility, serum IL-12 levels in CCR5^−/−^ and wild-type mice in both the C57BL/6 and C57BL/6x129 backgrounds were measured before infection and at days 3, 5, and 9 post-oral infection with T. gondii (Figure 7). We found a similar IL-12 serum response in CCR5^−/−^ and wild-type mice in the C57BL/6 background. Baseline levels were the same in wild-type and CCR5^−/−^ mice, and following infection serum IL-12 levels peaked on day 5 at 15–20 ng/ml in both wild-type and CCR5^−/−^ in the C57BL/6 background. Thus, in this genetic background the diminished NK cell response could not be accounted for by a blunted IL-12 response. However, as reported [21], CCR5^−/−^ mice in the C57BL/6x129 background had an approximately 50% reduction in the serum IL-12 response following T. gondii infection, with levels on day 9 PI of 12 ng/ml (± 3.2 ng/ml) in wild-type mice and 7.6 ng/ml (± 1.7 ng/ml) in CCR5^−/−^ mice (Figure 7B). Thus, in the C57BL/6x129 background, a blunted IL-12 response may have contributed to the increased parasite burden seen in these CCR5^−/−^ mice on day 14 (Figure 2C).

**Figure 8:**
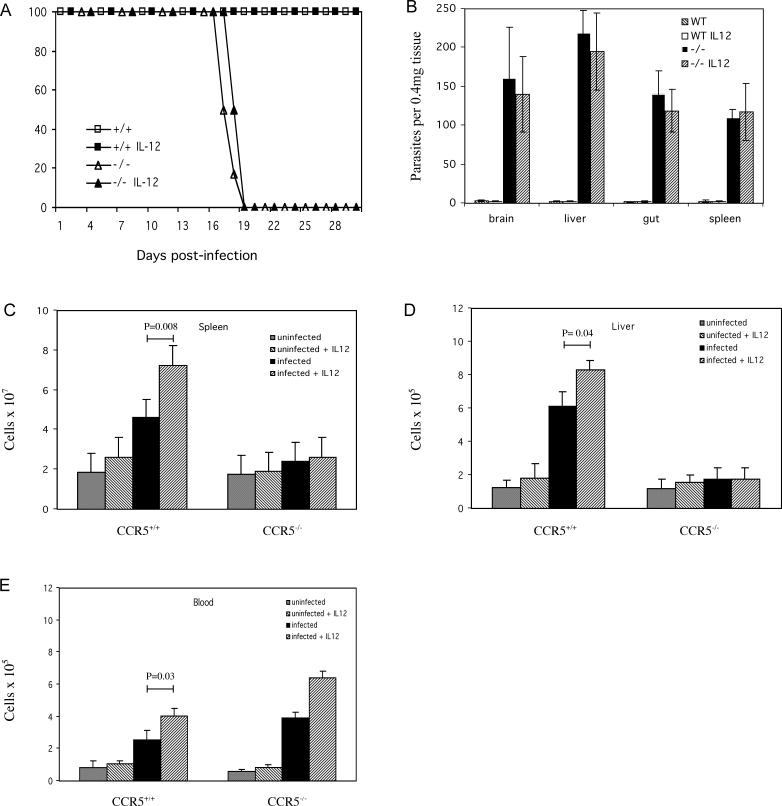
Survival and NK Cell Response following Exogenous IL-12 Treatment of CCR5^+/+^ and CCR5^−/−^ Mice (A and B) Effect of IL-12 administration on survival in the C57BL/6 x129 background. CCR5^−/−^ and CCR5^+/+^ mice (three mice per group) were treated with recombinant murine IL-12 (0.33 μg/mouse) daily intraperitoneally starting one day prior to infection with T. gondii. Uninfected controls treated with either IL-12 or saline were included in the study (six mice per group). Mice were challenged orally with 50 cysts and survival was monitored daily. Parasite load (B) was determined for mice described in (A). Mice were sacrificed on day 14 PI (three mice per group), and tissues (brain, liver, small intestine, and spleen) analyzed for parasite load by competitive DNA PCR. (C–E) Effect of exogenous IL-12 treatment on the NK cell response. CCR5^−/−^ and CCR5^+/+^ mice were infected orally with the 76K strain of T. gondii (three mice per group) and were treated with recombinant murine IL-12 (0.33 μg/mouse) via intraperitoneal route starting one day prior to infection. Uninfected controls treated with either IL-12 or saline were included in the study (three mice per group). At day 7 PI, single-cell suspensions from the spleens (C), and lymphocytes from liver (D) and blood (E) were prepared and cells were analyzed for the expression of the NK1.1 marker by FACS (experiment was performed twice with similar results).

To determine whether the increased susceptibility of CCR5^−/−^ mice in the C57BL/6x129 background was related to impaired IL-12 production, the effect of daily exogenous IL-12 therapy on mortality was determined (Figure 8A). IL-12 administration had no effect on the survival of CCR5^−/−^ mice, and the mortality and parasite load in these animals was indistinguishable from those in untreated CCR5^−/−^ mice (Figure 8A and 8B). The bioactivity of the cytokine was further tested by administering IL-12 to wild-type mice infected intraperitoneally with an LD_100_ dose of T. gondii tachyzoites. Treatment of these animals with daily exogenous IL-12 protected them from lethal infection (unpublished data), in agreement with a previous report [23].

To further establish that the effect of CCR5-deficiency on the NK cell response observed in T. gondii-infected mice in the C57BL/6x129 background occurred downstream of IL-12, we evaluated the NK cell tissue response in IL-12 reconstitution experiments in which we treated wild-type and CCR5^−/−^ mice with daily injections of IL-12 following T. gondii infection. As expected, IL-12 treatment increased NK cell numbers in the spleen and liver in infected wild-type mice compared to non-IL-12 treated wild-type controls (Figure 8C and 8D), demonstrating that IL-12 administered in this manner is biologically active, as has been previously reported [23]. In marked contrast, however, IL-12 had no effect on NK cell numbers in the spleen (Figure 8C) or liver (Figure 8D) of infected CCR5^−/−^ mice. In these mice, however, IL-12 increased the number of NK cells in the blood (Figure 8E), demonstrating that CCR5^−/−^ mice responded to IL-12 therapy, but were unable to recruit these mobilized NK cells into tissue. These data establish that the impaired immune response to T. gondii seen in CCR5^−/−^ mice is not solely the result of an impaired ability to generate IL-12, but is also the result of an inability to effectively mobilize NK cells into infected tissue.

### NK Cell Depletion Recapitulates the CCR5^−/−^ Phenotype

**Figure 9:**
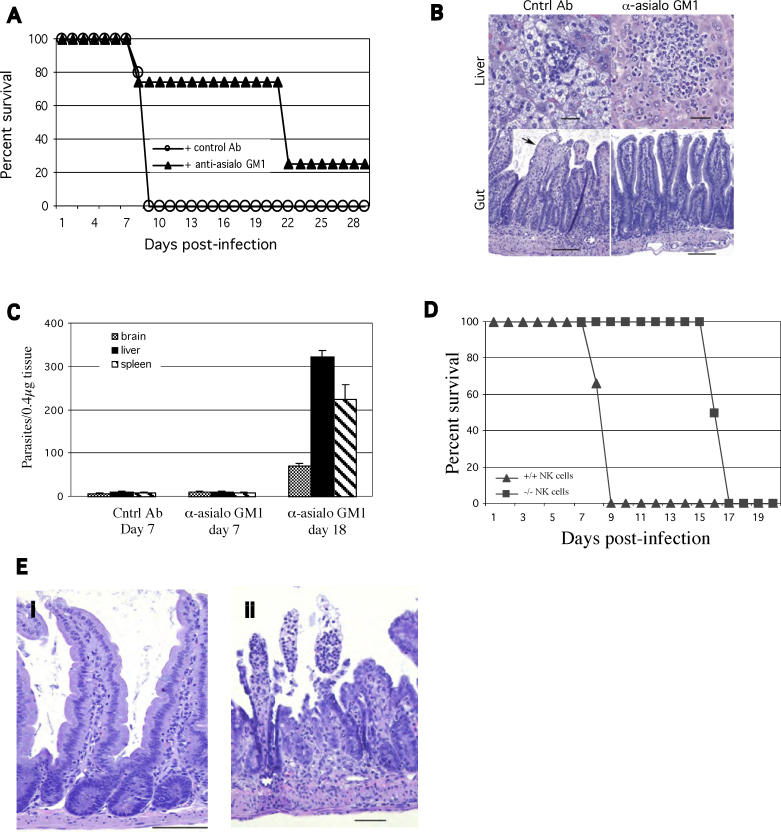
Effect of NK Cells on Survival following T. gondii Infection of C57BL/6 Mice (A–B) NK cell depletion of wild type. C57BL/6 mice were infected orally with 50 cysts. One day before and every day after infection mice were injected intraperitoneally with anti-asialo GM1 antibody. The treatment was continued for the duration of the experiment. Eight animals were treated per group, and data are representative of two separate experiments. Survival (A) and histopathology (B) of NK cell depleted and nondepleted T. gondii-infected CCR5^+/+^ C57BL6 mice. Photomicrographs of hematoxylin and eosin-stained liver and ileum of small intestine isolated from infected anti-asialo GM1 and control antibody-treated mice. NK cell-depleted mice had essentially normal small bowel morphology, and the livers showed an acute inflammatory response compatible with acute T. gondii infection. In nondepleted wild-type mice, the small bowel showed extensive, scattered, superficial necrosis, with acute inflammation, including polymorphonuclear cells in the lamina propria. The livers show marked, confluent fatty degeneration of hepatocytes, with small inflammatory nodules in the parenchyma consistent with a hyperimmune inflammatory response. Size bars: liver, 20 μm; small bowel 100 μm. The arrow indicates an area of superficial necrosis of epithelial cells. (C) Parasite load. Wild-type C57BL/6 mice were infected with 50 cysts orally and NK cell depletion performed as described above. At day 7 and 18 PI mice (three per group) were sacrificed and tissues (brain, liver, and spleen) analyzed for parasite load by competitive DNA PCR. (D–E) NK cell adoptive transfer. NK cells from CCR5^−/−^ and wild-type C57BL/6 mice were isolated and 10^7^ purified NK cells were injected to naïve CCR5^−/−^ mice. Recipients were subsequently challenged with 50 cysts of T. gondii. Six animals were treated per group, and data represent two separate experiments. Survival (D) and histopathology (E) of T. gondii-infected CCR5^−/−^ C57BL/6 treated with either CCR5^−/−^ or CCR5^+/+^ NK cells. Mice described above (two mice each) were sacrificed on day 9 and ileum isolated and stained with hematoxylin and eosin. In (E), small bowel of CCR5^−/−^ C57BL/6 recipients treated with CCR5^−/−^ NK cells (i) demonstrates preservation of the mucosa, as seen in large areas of the bowel wall. Bar, 100 μm. In contrast, in CCR5^−/−^ C57BL/6 recipients treated with wild-type NK cells (ii), the small bowel mucosa is characterized with superficial necrosis and with an inflammatory cell infiltrate of in the lamina propria, typical of hyperinflammatory response. Bar, 100 μm.

Our data suggest that CCR5 expression on NK cells is critical for NK cell trafficking into tissue in response to T. gondii infection, and that this impairment in NK cell trafficking into tissue explains the phenotype observed in CCR5^−/−^ mice. If this is the main reason for the observed phenotype, then it follows that NK cell depletion of wild-type mice should reveal a similar phenotype following T. gondii infection. We therefore depleted NK cells in wild-type C57BL/6 mice using anti-asialo GM1 antibody treatment to determine whether depletion of NK cell results in a reduced inflammatory response similar to CCR5^−/−^ mice in this genetic background. As shown in Figure 9A, while C57BL/6 wild-type mice treated with control antibody died by day 9 PI, the majority of NK cell-depleted mice survived until day 21 PI. To demonstrate that the protective effect of NK cell depletion on early mortality was the result of an attenuated tissue hyperinflammatory response, tissues were analyzed histopathologically (Figure 9B). As expected, mice treated with control antibody exhibited extensive necrosis of the ileum of small bowel, with acute inflammation, including polymorphonuclear cells in the lamina propria. The liver showed marked confluent fatty degeneration of hepatocytes, with small inflammatory nodules in the parenchyma (Figure 9B). In contrast, the small intestines of anti-asialo GM1-treated mice revealed no necrosis, with preservation of epithelial cells within the villi. Livers of these NK cell-depleted mice showed an acute inflammatory infiltrate, which was compatible with acute infection seen in T. gondii-infected mice that do not have a hyperimmune inflammatory response. To establish that mortality at the later time points in the NK cell-depleted mice was due to infection and not a tissue hyperimmune response, tissues (spleen, brain, and liver) from NK cell-depleted mice were analyzed for parasite burden. On day 7 PI, when control antibody-treated C57BL/6 mice were beginning to succumb to their infection but NK cell-depleted mice were not, parasite load was low in both groups. However, by day 18 PI, when NK cell-depleted mice were beginning to succumb to their infection, tissue parasite load in these mice had increased several hundred-fold (Figure 9C). All control antibody-treated infected mice died by day 10 PI and were therefore not available for analysis on day 18 PI. As a further control, we found that anti-asialo GM1 antibody treatment of uninfected mice had no effect on mortality (unpublished data). To determine whether the increased mortality and tissue hyperimmune response in C57BL/6 mice was the result of CCR5-mediated NK cell trafficking, we adoptively transferred wild-type and CCR5^−/−^ NK cells into CCR5^−/−^ mice followed by T. gondii challenge. As can be seen in Figure 9D, adoptive transfer of wild-type NK cells increased the susceptibility of CCR5^−/−^ C57BL/6 mice to *T. gondii,* and this was associated with a hyperimmune response in the small intestine (Figure 9E) and liver (unpublished data), which was not seen in CCR5^−/−^ mice that received CCR5^−/−^ NK cells.

**Figure 10:**
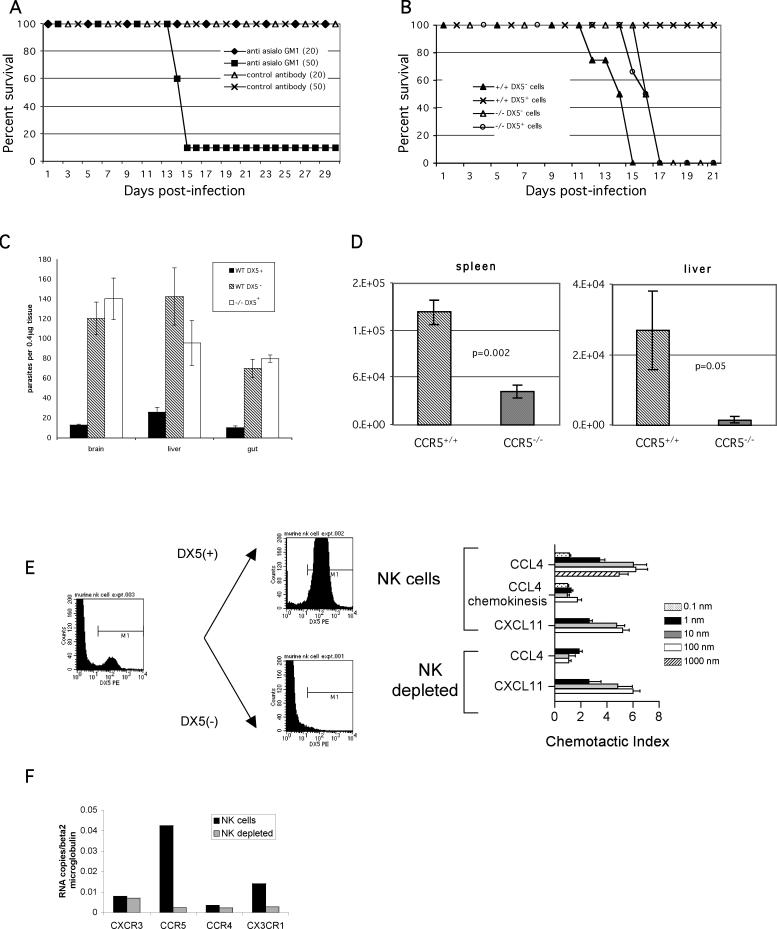
Effect of NK Cells on Survival following T. gondii Infection of C57BL/6x129 Mice and CCR5-Dependent Homing of NK cells (A) NK cell depletion of wild-type C57BL/6x129 mice. Mice were infected orally with 20 or 50 cysts of T. gondii. One day prior to infection and then three times a week for 2 wk, mice were injected intraperitoneally with anti-asialo GM1 antibody or a control rabbit IgG. Data represent two separate experiments (six mice per group). (B) Adoptive transfer of NK cells protects CCR5^−/−^ mice in the C57BL/6 x129 background against T. gondii infection. DX5^+^ cells from spleens of uninfected C57BL/6x129 wild-type and CCR5^−/−^ mice were isolated by magnetic separation. 1 × 10^7^ DX5^+^ NK cells and the same number of DX5^−^ splenocytes or an equal volume of saline were injected intravenously into naïve syngeneic C57BL/6x129 CCR5^−/−^ animals. At 48 h after transfer, animals were challenged orally with 50 cysts of T. gondii and survival monitored daily. The experiment was performed twice with similar results, and representative data are shown. (C) Parasite load. Mice described in (B) (three mice per group) were sacrificed on day 12 and tissues (brain, liver, and spleen) analyzed for parasite load by competitive DNA PCR. (D) CCR5-dependent homing of adoptively transferred NK cells. DX5^+^ CD45.2^+^ cells from uninfected CCR5^−/−^ and wild-type mice were isolated by affinity purification. 2 × 10^6^ purified (CD45.2) DX5^+^ cells were transferred to naïve congenic CD45.1 mice. One group (three mice per group) received DX5^+^ CD45.2^+^ cells from wild-type mice, while the other group was injected with DX5^+^ CD45.2^+^ cells isolated from CCR5^−/−^ mice. Recipients were subsequently challenged with 50 cysts orally and 3 d later animals were sacrificed. Splenocytes and hepatocytes isolated from the recipients were stained with FITC-labeled anti-CD45.2 antibody and number of transferred CD45.2^+^ cells determined by flow cytometry. (E) CCR5-dependent NK cell chemotaxis. NK cells respond to the CCR5 specific agonist CCL4 (MIP-1β). Purified DX5^+^ splenocytes (NK cells) and DX5-depleted splenocytes (NK cell-depleted) were analyzed in transwell chemotaxis assays. Chemotaxis and chemokinesis (control) of NK cells and NK-depleted splenocytes were tested at the indicated CCL4 (MIP-1β CCR5-specific agonist) and CXCL11 (I-TAC) (CXCR3-specific agonist) concentrations. Each data point is the mean ± standard deviation of triplicate measurements and represents three independent experiments. (F) DX5^+^ NK cells express CCR5 mRNA. DX5^+^ NK cells and DX5^−^ C57BL/6 splenocytes were analyzed for chemokine receptor expression profile by quantitative real-time PCR. These data represent three experiments.

To determine whether absence of NK cells can also affect the outcome of T. gondii infection in the C57BL/6 x129 background, wild-type and CCR5^−/−^ mice in this background were depleted of NK cells using an anti-asialo GM1 antibody treatment. Depletion of NK cells resulted in mice that had a dose-dependent increase in mortality following infection. Thus, while NK cell depletion had no effect on survival following an infection with 20 cysts, infection with 50 cysts resulted in 90% mortality in NK cell-depleted mice (Figure 10A). No increase in mortality was observed in mice treated with a control antibody (Figure 10A). Thus, depletion of NK cells recapitulated the phenotypes seen in CCR5^−/−^ mice in both the C57BL/6 and C57BL/6x129 backgrounds following T. gondii infection.

### Adoptive Transfer of Wild-Type NK Cells Confers Resistance in CCR5^−/−^ Mice

To further determine whether CCR5 expression on NK cells was critical for host resistance to *T. gondii,* adoptive transfer studies were performed using CCR5^−/−^ mice in the C57BL/6x129 background as the recipients, and the following syngeneic cell preparations as donors: wild-type and CCR5^−/−^ splenocytes, NK cell-depleted wild-type splenocytes, and purified wild-type and CCR5^−/−^ NK cells. Spleen cells from uninfected wild-type and CCR5^−/−^ mice were depleted of NK cells by magnetic separation using the NK marker DX5, which was also used to positively select NK cells. DX5 expression has been reported to be restricted to NK cells and a minority of activated T cells and has been used to purify NK cells to evaluate their functional activities [24]. Since we isolated DX5^+^ cells from spleens harvested from naïve mice, it is unlikely that there were significant numbers of activated T cells in the donor cell population. CCR5^−/−^ mice in the C57BL/6x129 background were injected intravenously with 10^7^ DX5^+^ NK cells or DX5^−^ splenocytes or equal volume of saline. At 48 h after transfer, recipients were orally challenged with 20 or 50 cysts. Infection with 20 cysts produced no mortality in any group (unpublished data). However, infection with 50 cysts induced 100% mortality by day 16 PI in mice that received DX5-depleted splenocytes or saline (Figure 10B). In contrast, mice that received DX5^+^ wild-type cells controlled their infection and survived. Conversely, DX5^+^ cells from CCR5^−/−^ donors were unable to protect recipient mice against a high dose infection (Figure 10B). To investigate whether the survival of recipients of DX5^+^ wild-type NK cells was the result of decreased parasite replication, we determined the tissue parasite load following adoptive transfer of wild-type DX5^+^ and DX5^−^ cells and CCR5^−/−^ DX5^+^ cells (Figure 10C). We found reduced parasite loads in the brain, liver, and small intestine of mice that received CCR5^+/+^ DX5^+^ cells but not CCR5^−/−^ DX5^+^ cells or CCR5^+/+^ DX5^−^ cells. These data demonstrate that CCR5 function on NK cells is critical for host resistance to T. gondii.

### Impaired Homing of Adoptively Transferred CCR5^−/−^ NK Cells

To establish that CCR5 plays an important role in NK cell trafficking, congenic (CD45.1/CD45.2) C57BL/6 mice were used for adoptive transfer experiments. Purified NK cells (2 × 10^6^ DX5^+^) isolated from naïve wild-type and CCR5^−/−^ CD45.2 mice were transferred into congenic CD45.1 mice by intravenous injection. At 48 h post-transfer, recipient mice were challenged orally with 50 cysts of T. gondii. Recipients were sacrificed 3 d after the challenge and organs (spleen and liver) were analyzed by FACS for the presence of CD45.2 cells. In comparison to transferred wild-type NK cells, transferred CCR5^−/−^ NK cells had a significantly reduced ability to migrate into the spleen (*p* = 0.002) and liver (*p* = 0.05) of recipient wild-type mice (Figure 10D). These data demonstrate that CCR5 expression on NK cells plays an important role their homing to *T. gondii-*infected organs.

### CCR5 Agonists Induce NK Cell Chemotaxis

Our data suggest that NK cell recruitment in vivo in response to T. gondii infection is mediated by CCR5 expressed on NK cells. CCR5 has been reported to be highly expressed on murine NK cells [14]. To directly test whether CCR5 is functional on NK cells, we purified NK cells from the spleens of mice using DX5, an NK cell marker. We found that DX5^+^ NK cells had a robust chemotaxis response to CCL4/MIP-1β, a CCR5-specific agonist (Figure 10E). In contrast, DX5^−^ splenocytes had no chemotactic response to CCL4, but responded robustly to the positive control CXCR3 agonist CXCL11/I-TAC. These data demonstrate that CCR5 is functional on NK cells and plays an important role in NK cell trafficking in vivo. Furthermore, we analyzed these DX5^+^ NK cells and DX5^−^ splenocytes for chemokine receptor mRNA expression by quantitative real-time PCR. As shown in Figure 10F, although NK cells and NK-depleted cells express similar mRNA levels of CXCR3, NK cells were enriched for CCR5 mRNA compared to NK-depleted cells, confirming the chemotaxis data, which demonstrated functional CCR5 expression on NK cells. Moreover, NK cells were enriched for CX3CR1, but had low CCR4 levels, a profile consistent with that of human NK cells [12]

## Discussion

The findings presented here demonstrate that CCR5 is a critical NK cell homing receptor, controlling NK cell trafficking into tissues in a murine model of T. gondii infection. While CCR5-deficient C57BL/6 mice had an early burst of CCR5 ligand production, their inability to mount a tissue NK cell response resulted in decreased tissue inflammation characterized by diminished IFNγ and subsequent chemokine expression in the tissues. This impaired inflammatory response protected the mice from the lethal hyperimmune response evoked by T. gondii in the C57BL/6 background. As a result, however, CCR5^−/−^ mice had an increased parasite burden and thus succumbed to infection when challenged with high doses of T. gondii cysts in a genetic background (C57BL/6x129) that is not susceptible to the hyperimmune response. The impaired immune response to acute T. gondii infection in CCR5^−/−^ was characterized by deficient recruitment of NK cells into the tissue. Adoptive transfer of CCR5^+/+^ NK cells into CCR5^−/−^ C57BL/6x129 mice restored their natural resistance to T. gondii infection and demonstrated the CCR5 dependence for NK cell trafficking into infected tissue. These studies establish CCR5 as a critical receptor for NK cell trafficking and innate host defense.

NK cells are produced in the bone marrow and circulate in the blood. In response to infection or inflammation, NK cells leave the circulation and enter the tissues where they play an essential role in early innate host defense. The molecular signals that control the recruitment of NK cells into tissues are not understood. Our study clearly establishes CCR5 as an important receptor guiding NK trafficking into T. gondii-infected tissue. In orally infected animals we found markedly reduced numbers of CCR5^−/−^ NK cells in spleen and liver, tissues in which we observed an early induction of CCR5 ligand expression. We did not observe an increase in NK cell influx into the small intestine of either wild-type or CCR5^−/−^ mice. Interestingly, we found no significant expression of CCR5 ligands in this tissue following infection until day 7; even then it was much less than that observed in the spleen, lung, and liver. We also found that CCR5^−/−^ NK cells accumulated in the blood and mesenteric lymph node of infected animals. It was recently shown that CXCR3 plays an important role in NK cell trafficking into inflamed lymph nodes [25]. We suspect that NK cells can enter the infected lymph node via CXCR3 but cannot move out into the tissue in the absence of CCR5 signaling.

Adoptive transfer experiments confirmed that CCR5 expression was required for NK cell trafficking into infected tissue. CCR5^−/−^ mice have normal NK cell numbers in the uterus, which may suggest a difference in the control of homeostatic NK cell trafficking versus NK cell trafficking in response to an infection or differences in organ-specific NK cell trafficking [26]. CCR5 has been shown to be homogeneously highly expressed on murine NK cells (DX5^+^, CD11b^+^, CD4^−^, CD8^−^) [14], and we found that CCR5 was markedly enriched on DX5^+^ splenocytes compared to DX5^−^ splenocytes, and that DX5^+^ splenocytes migrated in response to a gradient of CCL4/MIP-1β, a CCR5-specific agonist, while DX5^−^ splenocytes did not. Expression of CCR5 on murine NK cells may, however, differ from human NK cells, in which CCR5 is highly expressed only on a subset (CD56^+^CD16^−^) of NK cells [12]. However, CCL3/MIP-1α has been shown to chemoattract human NK cells [27], and CCL3/MIP-1α was identified as the major NK cell chemoattractant released from cytotrophoblasts [28]. These latter studies suggest that CCR5 may also play an important role in human NK cell trafficking.

Our study demonstrates an important role for CCR5 in NK cell trafficking to T. gondii-infected liver and spleen. In doing so, our data also revealed an unappreciated role of the NK cell in regulating tissue inflammation induced by T. gondii. It has been generally believed that IFNγ-producing CD4^+^ T cells in T. gondii-infected mice are the primary mediators of the hyperinflammatory response [20]. However, recent studies in our laboratory have demonstrated that CCR1^−/−^ mice in the C57BL/6 background had a defect in neutrophil trafficking, which also resulted in a reduced hyperimmune response following T. gondii infection [3]. Thus, it appears that in addition to CD4^+^ T cells, innate immune cells such as NK cells and neutrophils also contribute to the hyperimmune response in T. gondii-infected animals. The important role of NK cells during early *Toxoplasma* infection has been well described. Administration of IL-12 to normal immunocompetent mice enabled them to tolerate lethal infective doses of T. gondii [23]. In the same studies, depletion of NK cells reversed the protective effect of IL-12. Similarly, IL-12 treatment of SCID mice, which lack T and B cells, enabled them to survive longer with a T. gondii infection [6]. It is believed that NK cells are an important early source of IFNγ during acute T. gondii infection [8]. Our current data support these findings regarding an important role for NK cells during acute *Toxoplasma* infection. Absence of an optimal NK cell response at the site of infection in the CCR5^−/−^ mice lead to a defective clearance of parasites, resulting in increased mortality. Although less critical than T cells, which are needed for long-term host survival [29], NK cells play an essential role in host survival following challenge with a high parasite load. In the present study, we observed that CCR5^−/−^ mice on the C57BL/6x129 background, when challenged orally with 20 cysts, could survive the infection. However, when the dose was increased to 50 cysts, optimal NK cell migration to infected sites was needed, without which the host was unable to survive. Furthermore, transfer of splenocytes as well as purified NK cells from uninfected wild-type mice protected CCR5^−/−^ mice against this high challenge, while the transfer of NK cell-depleted splenocytes did not confer protection in these mice. These data demonstrate that in the presence of high doses of *T. gondii,* NK cells are required for parasite clearance, and their absence ultimately results in elevated parasite burden in the tissues, which leads to increased mortality.

CCR5 has been implicated in the host response to *T. gondii.* The CCR5 ligands CCL3/MIP-1α and CCL4/MIP-1β have been shown to be induced in splenic endothelial cells and dendritic cells within 2 h of the injection of soluble T. gondii antigen [21]. CCL3 and CCL4 were also shown to be induced in the small intestine 7 d following T. gondii infection [30]. In this latter study, adoptive transfer of CCR5^−/−^ CD8α^+^ intraepithelial lymphocytes from T. gondii-infected mice into naive animals had reduced ability to migrate into the intestine of recipient mice following infection. As intraepithelial lymphocytes are unconventional lymphocytes, it is difficult to compare these studies with our observations and we did not specifically evaluate lymphocyte populations in the small intestine. Furthermore, cyclophilin-18, a soluble T. gondii protein, was shown to induce the production of IL-12 through activation of CCR5 [31]. However, neutrophils containing prestored IL-12 are also an important source of IL-12 [2], which may be especially important in the CCR5-deficient mice. Our study does not directly address this issue, but we found that CCR5 must also act downstream of IL-12. We observed that CCR5^−/−^ mice in the C57BL/6 background had an equivalent increase in serum IL-12 levels following T. gondii infection. Thus, the phenotype that we observed in CCR5^−/−^ mice in this background—a blunted hyperimmune response and a decrease in NK cell recruitment into tissues—could not be explained by a blunted IL-12 response. We did, however, confirm the findings of Aliberti et al. [21], who reported an approximately 50% decrease in serum IL-12 levels in CCR5^−/−^ C57BL/6x129 mice following T. gondii infection. Thus CCR5-dependent IL-12 production may play a role in controlling parasite replication in CCR5^−/−^ mice in C57BL/6x129 background. However, we do not believe that this partial reduction in serum IL-12 fully accounts for the dramatic phenotype we observed for CCR5^−/−^ mice in this background. We could not restore resistance of CCR5^−/−^ mice to T. gondii infection with exogenous IL-12 treatment. Furthermore, exogenous IL-12 did not reverse the defect in NK cell trafficking we observed in CCR5^−/−^ mice, even though IL-12 augmented NK numbers recovered from the spleen of uninfected and infected wild-type mice. Furthermore, exogenous IL-12 increased the resistance of wild-type mice to a lethal T. gondii challenge. Thus, taken together, these data support an additional and major role for CCR5 downstream of IL-12 production in the recruitment of NK cells into T. gondii-infected tissue.

The chemokine system is a complex cytokine network that controls the trafficking of leukocytes. A functional appreciation for the roles of individual members of this superfamily has come from studies using genetically deficient mice or spontaneous mutations in humans. CCR5 is the best example of this paradigm, with the generation of CCR5-deficient mice and the identification of a CCR5 null mutation in the human population. CCR5-deficient mice have been described and characterized as impaired in their ability to handle infection with *Listeria* [32] and influenza A virus [33], and as having diminished IFNγ production in response to Leishmania donovani infection [34]. These mice have also been shown to be protected from lipopolysaccharide-induced endotoxemia [32] and dextran-sulfate injury of the bowel [35].

Humans with a naturally occurring null mutation in CCR5 (CCR5-Δ32) are resistant to HIV-1 infection, and lymphocytes from these individuals were resistant to “M”-tropic HIV-1 entry [36–40]. Since these important discoveries, it has been realized that these CCR5 null individuals, which represent about 1% of persons of European decent, would be of interest in terms of their susceptibility to infectious and inflammatory diseases. Studies examining disease susceptibility in these CCR5-Δ32 homozygotes are ongoing and some studies have already been published on rheumatoid arthritis [41–43], systemic lupus erythematosis [41], inflammatory bowel disease [44], and multiple sclerosis [45,46] in these persons. To date, of all these conditions only rheumatoid arthritis appears to be affected in CCR5 individuals; a slight reduction in severity was observed. However, CCR5-Δ32 homozygotes are ideal “models” in which to study this receptor in the function of NK cells and in the host response to pathogens such as T. gondii.

Our study also raises concern about the potential complication of impaired NK cell function for the clinical use of CCR5 antagonists currently being developed as therapeutics for HIV-1 and inflammatory diseases. This may be of particular concern for HIV-1 infected patients coinfected with T. gondii or for those who acquire an acute T. gondii infection while taking a CCR5 antagonist. Our results suggest that the blockade of CCR5 through the use of antagonists may prevent NK cell trafficking to tissue sites of infection following acute T. gondii and, thus, affect immune control of T. gondii.

Our study highlights two important points regarding the biology of chemokines and their receptors. First, although in vitro studies have implied the existence of redundancy in the chemokine system, as evidenced by the fact that NK cells express multiple chemokine receptors and respond to multiple chemokines, studies such as ours have revealed that individual chemokines and chemokine receptors play important, nonredundant roles in vivo. Second, our study dramatically underscores the importance of genetic background when examining the effects of a chemokine or chemokine receptor gene in a complex biological process, such as host defense against pathogens. In the pure C57BL/6 background, the absence of CCR5 was protective, because it led to a diminished hyperimmune response, which in the C57BL/6 background can be lethal. However, in the mixed C57BL/6x129 background, which is not susceptible to the hyperimmune response, and can otherwise withstand high doses of infection, this diminished inflammatory response led to increased mortality from uncontrolled parasite growth. Thus, the absence of CCR5 and the resultant impaired recruitment of IFNγ-secreting NK cells has a different outcome on the host response to T. gondii depending on the genetic background.

Based on our current data and prior studies [15,16,47], a picture is emerging regarding the critical role of chemokines in linking the innate and acquired immune response to pathogens such as T. gondii. T. gondii infection induces an early and rapid burst in CCL3 and CCL4 production in specific infected tissue, such as the spleen, liver, and lung. CCL3 and CCL4 recruit IFNγ-secreting NK cells into infected tissue in a CCR5-dependent manner. IFNγ induces the production of chemokines, including CXCL10, from resident tissue cells that attract antigen-specific CD4^+^ and CD8^+^ lymphocytes into infected tissue to establish organ-specific immunity [17]. Thus, analogous to antiviral defenses described for cytomegalovirus [15,16,47], the host response to T. gondii requires a chemokine-to-cytokine-to-chemokine cascade. This likely represents a general principle that links the innate and acquired immune response to intracellular pathogens, which is essential for protective immunity.

Based on these findings, we conclude that the presence of CCR5 is required for the induction of the inflammatory response during T. gondii infection. The absence of this response, although initially advantageous in the C57BL/6 background because of the absence of the hyperimmune response, leads to uncontrolled parasite multiplication and ultimate death of the infected host in the C57BL/6x129 background.

## Materials and Methods

### Mice and parasites.

CCR5^−/−^ mice in the C57BL/6x129 background were generated as previously described and wild-type littermates were used as controls [48]. The CCR5^−/−^ transgene was also back-crossed eight generations into the C57BL/6 background. Wild-type C57BL/6 used for the backcross were used as controls. Congenic CD45.1 mice were obtained from Jackson laboratories (Bar Harbor, Maine, United States). Mice were challenged perorally with the 76K strain of T. gondii (kindly provided by Dr. Daniel Bout, Tours, France). This strain is maintained by continuous oral passage of cysts.

### Quantitation of parasite burden.

Tissues (small intestine, spleen, liver, lung, and brain) from T. gondii-infected animals were collected at days 7 or 15 PI*.* DNA was extracted from tissues using the QiaAmp tissue kit (Qiagen, Valencia, California, United States), and 400 ng of each sample was analyzed by quantitative PCR. Amplification of parasite DNA was performed using primers specific for a 35-fold repetitive sequence of the *T. gondii B1* gene (5′-GGAACTGCATCCGTTCATGAG-3′ and 5′-TCTTTAAAGCTTCGTGGT C-3′), which is found in all known parasite strains [49]. A 134 bp competitive internal standard containing the same primer template sequences as the 194 bp *B1* PCR fragment was also synthesized [50]. Amplification of this 194 bp segment of the *B1* gene and the 134 bp segment of the internal standard was performed using in a 50 μl reaction mixture containing 1.25 units of AmpliTaq DNA polymerase, 1× buffer (Perkin Elmer, Wellesley, California, United States), 0.2 mM each of dGTP, dATP, dTTP, and dCTP, and 0.4 μM of each *B1* primer. For each reaction a known amount of DNA from the tissues was amplified with varying amounts of the internal standard. Parasite loads were estimated by comparison to the internal controls. To determine the parasite load in infected tissues, PCR was performed under the same conditions using a known number of parasites. The level of internal control was calculated per parasite [50].

### Histopathological analysis.

Tissues from infected CCR5^−/−^ animals and control C57BL/6 animals were fixed in 10% buffered formalin, paraffin processed, and used to prepare 5 μm histological sections. Sections were stained with hematoxylin and eosin and photographed on an Olympus Van Ox microscope (Olympus, Tokyo, Japan) with Kodak Elite 100 film. The resulting images were digitized with a Polaroid Sprint scanner and processed using Adobe Photoshop software.

### Phenotypic analysis.

Single-cell suspensions of splenocytes were prepared using standard procedures, and hepatic lymphocytes were isolated using a modification of published methods [51]. In brief, livers were perfused with sterile PBS prior to excision to remove blood elements. Excised livers were minced and passed through a mesh screen. The cells were washed with PBS and subjected to density gradient centrifugation on Percoll (Sigma, St. Louis, Missouri, United States) to isolate the lymphocyte population. For isolating peripheral blood lymphocytes, blood was collected by cardiac puncture in a microcentrifuge tube containing heparin. About 500–700 μl of blood was obtained by this method and lysis of red blood cells was performed using Red Cell Lysis Buffer (Sigma). The cell pellet was resuspended in PBS containing 1% BSA. Splenocytes, mesenteric lymph node cells, and hepatic lymphocytes were suspended in PBS containing 10% FCS, and all the samples were counted in a hemocytometer prior to staining with the fluorochrome-conjugated antibodies anti-CD3-FITC, anti-CD8-FITC, anti-NK1.1-PE, and anti-CD45.2-FITC (clone 104) (all from BD PharMingen, San Diego, California, United States) and anti-CD4-Red613 (GIBCO BRL, Grand Island, New York, United States). Two- and three-color flow cytometry was performed on a FACscan cytometer (Becton Dickinson, San Jose, California, United States) and the data were analyzed using Lysys software (Hewlett-Packard, Palo Alto, California, United States). The absolute number of positive cells was calculated as follows: Total number of cells recovered from organ × percentage of cells staining positive/100.

### Proliferation assay.

CD4^+^ and CD8^+^ T cells were purified from splenocytes by positive selection using microbeads (Miltenyi Biotec, Bergisch Gladbach, Germany). The purity of the cells exceeded 95% as determined by FACS analysis. Purified cells were cultured in 96-well plates at the concentration of 2 × 10^5^ cells/well in 200 μl. Cells were stimulated either with ConA (2.5 μg/ml; Sigma) or *Toxoplasma* lysate antigen (10 μg/ml) in the presence of 1 × 10^6^ irradiated feeder cells (3,000 rads) obtained from syngeneic mice. After 72 h incubation at 37 °C in 5% CO_2_, cells were pulsed with ^3^H thymidine (0.5 μCi/well) for 8 h to determine rate of DNA synthesis. Pulsed cells were harvested on a glass filter and dried, and radioactive thymidine incorporation was measured by liquid scintillation.

### IFNγ assay.

CD4^+^ and CD8^+^ T cells from CCR5^−/−^ and wild-type mice were purified from spleens on day 7 PI. Purified cells were cultured at 1 × 10^6^ cells/well in 24-well plates in the presence of 1 × 10^6^ irradiated feeder cells from naïve syngeneic mice. Cultures were stimulated with 10 μg/ml of *Toxoplasma* lysate antigen, and supernatants were collected after 72-h stimulations. Supernatants were analyzed for IFNγ by ELISA using mouse IFNγ immunoassay kit (R&D Systems, Minneapolis, Minnesota, United States).

### Intracellular IFNγ staining.

IFNγ production by T cells was evaluated by intracellular staining as previously described [52] using the Cytofix/CytoPerm kit (BD PharMingen, San Jose, CA). Wild-type and CCR5^−/−^ mice (aged 5–8 wk) were infected orally with 20 cysts of T. gondii. At day 7 PI, spleens were disrupted between glass slides, and red blood cells were removed by lysis with Red Blood Cell Lysing Buffer (Sigma). Cells were stimulated in 96 well plates with 10 ng/ml phorbol 12-myristate 13-acetate (Sigma), 500 ng/ml of ionomycin (Sigma), and 2 μM monensin (Golgistop; BD PharMingen) for 4 h at 37 °C in 5% CO_2_. After stimulation, cells were surface-stained with FITC anti-CD8α, or PE-Cy5 anti-CD4 (eBioscience, San Jose, California, United States). Intracellular staining was then performed using anti-IFNγ or an isotype-matched control antibody conjugated with PE (eBioscience). Samples were analyzed by FACS.

### Quantitative PCR analysis.

Quantitative PCR analysis was performed on total RNA isolated from lung, liver, small intestine, and spleen of CCR5^−/−^ and wild-type control mice (females aged 5–6 wk) infected orally with 15–50 cysts of 76K strain of T. gondii. Tissues were harvested 3, 5, and 7 days PI. Total RNA was extracted from tissue using Trizol (Invitrogen, Carlsbad, California, United States) or from magnetic bead purified cells using the RNeasy kit (Qiagen). After DNase I (Invitrogen) treatment, total RNA from each sample was used as a template for the reverse-transcription reaction. cDNA was synthesized using oligo(dT)_15_ and random hexamers. Oligonucleotide primers were designed using Primer Express software (PE Biosystems, Foster City, California, United States). QPCR was performed as described, using the Mx4000 Multiplex Quantitative PCR System (Stratagene, La Jolla, California, United States) [53]. Emitted fluorescence for each reaction was measured during the annealing/extension phase, and amplification plots were analyzed using the MX4000 software version 3.0 (Stratagene). Quantity values (i.e., copies) for gene expression were generated by comparison of the fluorescence generated by each sample with standard curves of known quantities, and the calculated number of copies divided by the number of copies of the constitutively active gene β2-microglobulin or GAPDH. Primers sets used are posted on our laboratory web site at http://www.immunologynet.org.

### Serum IL-12 p40/p70 ELISA.

Serum was collected via tail bleed from wild-type control and CCR5^−/−^ mice in both the C57BL/6 and C57BL/6x129 background either before infection or at days 3, 5, and 9 post oral infection with 50 cysts of 76K strain of T. gondii. IL-12 p40/p70 ELISA was performed on all samples using an immunoassay kit (Biolegend, San Diego, California, United States).

### Exogenous IL-12 administration.

Each mouse was injected intraperitoneally with 0.33 μg of recombinant murine IL-12 (Genetics Institute, Cambridge, Massachusetts, United States) 1 d prior to infection and then every day thereafter. Control mice were injected with an equal volume of saline. At day 7 PI, animals were sacrificed, single-cell suspensions from the spleens were prepared, and the cells were analyzed for the expression of NK1.1 marker by flow cytometry.

### NK cell depletion.

To deplete NK cells, mice were injected with 50 μg of anti-asialo GM1 antibody (Wako Pure Chemical, Richmond, Virginia, United States) intraperitoneally starting 1 d prior to infection. Treatment was continued three times a week for a period of 2 wk and once weekly there after.

### NK cell purification and chemotaxis.

NK cells were purified from C57BL/6 or BALB/c mice as described [54]. Briefly, unactivated splenocytes were incubated with anti-mouse CD16/CD32 (BD Pharmingen) and then stained with PE-DX5 mAb (BD Pharmingen), followed by incubation with magnetic microbeads coated with anti-PE-Ab (Miltenyi Biotec). DX5^+^ cells were isolated by MACS magnetic cell sorting (Miltenyi Biotec). NK-depleted splenocytes were suspended in RPMI with 1% bovine serum albumin and loaded in triplicate into a transwell chemotaxis apparatus (ChemoTx, Neuroprobe, Gaithersburg, Maryland, United States). Chemokines were placed in the lower wells at various concentrations and, as a control for chemokinesis, in the upper and lower wells at the same concentration. After incubation for 2 h at 37 °C, cell counts were determined in the lower wells by manual counting or with Cyquant fluorescent dye (Molecular Probes, Eugene, Oregon, United States) in a Cytofluor Multi-Well Plate Reader Series 4000 (Applied Biosystems, Foster City, United States).

### NK cell transfer into CCR5^−/−^ mice.

Naïve splenocytes from C57BL/6 x129 mice were collected as described above. Spleen cells were depleted of CD4^+^ T cells, CD8^+^ T cells, and B cells by magnetic sorting. The remaining cells were positively selected using a DX5 mAb (BD Pharmingen) and MACS (Miltenyi Biotec). DX5^−^ cells were isolated by negative selection from naïve splenocytes using anti-DX5 mAb and a magnetic column. Less than 1% of the DX5^−^ fraction stained positive for NK1.1^+^ as determined by FACS analysis.

### Statistical analysis.

Results of experimental studies are reported as mean ± standard deviation. Differences were analyzed using Student's t-test. A *p*-value less than 0.05 was regarded as significant.

## Abbreviations

IFNγ: interferon γ
IL: interleukin
NK: natural killer
PI: postinfection
QPCR: quantitative PCR

## References

[ppat-0020049-b001] Sher A, Denkers E, Gazzinelli R (1995). Induction and regulation of host cell-mediated immunity by Toxoplasma gondii. Ciba Found Symp.

[ppat-0020049-b002] Bliss SK, Butcher BA, Denkers EY (2000). Rapid recruitment of neutrophils containing prestored IL-12 during microbial infection. J Immunol.

[ppat-0020049-b003] Khan IA, Murphy PM, Casciotti L, Schwartzman JD, Collins J (2001). Mice lacking the chemokine receptor CCR1 show increased susceptibility to Toxoplasma gondii infection. J Immunol.

[ppat-0020049-b004] Suzuki Y, Orellana M, Schreiber R, Remington J (1988). Interferon-gammon: The major mediator of resistance against Toxoplasma gondii. Science.

[ppat-0020049-b005] Suzuki Y, Remington J (1988). Dual regulation of resistance against Toxoplasma gondii infection by Lyt-2^+^ and Lyt-1^+^, L3T4^+^ T cells in mice. J Immunol.

[ppat-0020049-b006] Gazzinelli RT, Hieny S, Wynn TA, Wolf S, Sher A (1993). Interleukin 12 is required for the T-lymphocyte-independent induction of interferon gamma by an intracellular parasite and induces resistance in T-cell-deficient hosts. Proc Natl Acad Sci U S A.

[ppat-0020049-b007] Denkers EY, Gazzinelli RT, Martin D, Sher A (1993). Emergence of NK1.1^+^ cells as effectors of IFN-gamma dependent immunity to Toxoplasma gondii in MHC class I-deficient mice. J Exp Med.

[ppat-0020049-b008] Hunter CA, Subauste CS, Van Cleave VH, Remington JS (1994). Production of gamma interferon by natural killer cells from Toxoplasma gondii-infected SCID mice: Regulation by interleukin-10, interleukin-12, and tumor necrosis factor alpha. Infect Immun.

[ppat-0020049-b009] Boehm U, Klamp T, Groot M, Howard JC (1997). Cellular responses to interferon-gamma. Annu Rev Immunol.

[ppat-0020049-b010] Akiyama K, Yokota K, Kagawa S, Shimbara N, Tamura T (1994). cDNA cloning and interferon gamma down-regulation of proteasomal subunits X and Y. Science.

[ppat-0020049-b011] Inngjerdingen M, Damaj B, Maghazachi AA (2000). Human NK cells express CC chemokine receptors 4 and 8 and respond to thymus and activation-regulated chemokine, macrophage-derived chemokine, and I-309. J Immunol.

[ppat-0020049-b012] Campbell JJ, Qin S, Unutmaz D, Soler D, Murphy KE (2001). Unique subpopulations of CD56^+^ NK and NK-T peripheral blood lymphocytes identified by chemokine receptor expression repertoire. J Immunol.

[ppat-0020049-b013] Inngjerdingen M, Damaj B, Maghazachi AA (2001). Expression and regulation of chemokine receptors in human natural killer cells. Blood.

[ppat-0020049-b014] Mack M, Cihak J, Simonis C, Luckow B, Proudfoot AE (2001). Expression and characterization of the chemokine receptors CCR2 and CCR5 in mice. J Immunol.

[ppat-0020049-b015] Salazar-Mather TP, Orange JS, Biron CA (1998). Early murine cytomegalovirus (MCMV) infection induces liver natural killer (NK) cell inflammation and protection through macrophage inflammatory protein 1alpha (MIP-1alpha)-dependent pathways. J Exp Med.

[ppat-0020049-b016] Salazar-Mather TP, Hamilton TA, Biron CA (2000). A chemokine-to-cytokine-to-chemokine cascade critical in antiviral defense. J Clin Invest.

[ppat-0020049-b017] Khan I, MacLean JA, Lee F, Casciotti L, DeHaan E (2000). The IP-10 chemokine is critical for effector T cell trafficking and host survival in Toxoplasma gondii infection. Immunity.

[ppat-0020049-b018] McLeod R, Skamene E, Brown CR, Eisenhauer PB, Mack DG (1989). Genetic regulation of early survival and cyst number after peroral Toxoplasma gondii infection of A × B/B × A recombinant inbred and B10 congenic mice. J Immunol.

[ppat-0020049-b019] Khan IA, Schwartzman JD, Matsuura T, Kasper LH (1997). A dichotomous role for nitric oxide during acute Toxoplasma gondii infection in mice. Proc Natl Acad Sci U S A.

[ppat-0020049-b020] Liesenfeld O, Kosek J, Remington JS, Suzuki Y (1996). Association of CD4^+^ T cell-dependent, interferon-gamma-mediated necrosis of the small intestine with genetic susceptibility of mice to peroral infection with Toxoplasma gondii. J Exp Med.

[ppat-0020049-b021] Aliberti J, Reis e Sousa C, Schito M, Hieny S, Wells T (2000). CCR5 provides a signal for microbial induced production of IL-12 by CD8 alpha^+^ dendritic cells. Nat Immunol.

[ppat-0020049-b022] Trinchieri G (1998). Proinflammatory and immunoregulatory functions of interleukin-12. Int Rev Immunol.

[ppat-0020049-b023] Khan IA, Matsuura T, Kasper LH (1994). Interleukin-12 enhances murine survival against acute toxoplasmosis. Infect Immun.

[ppat-0020049-b024] Tomasello E, Desmoulins PO, Chemin K, Guia S, Cremer H (2000). Combined natural killer cell and dendritic cell functional deficiency in KARAP/DAP12 loss-of-function mutant mice. Immunity.

[ppat-0020049-b025] Martin-Fontecha A, Thomsen LL, Brett S, Gerard C, Lipp M (2004). Induced recruitment of NK cells to lymph nodes provides IFN-gamma for T(H)1 priming. Nat Immunol.

[ppat-0020049-b026] Chantakru S, Kuziel WA, Maeda N, Croy BA (2001). A study on the density and distribution of uterine natural killer cells at mid pregnancy in mice genetically-ablated for CCR2, CCR 5 and the CCR5 receptor ligand, MIP-1 alpha. J Reprod Immunol.

[ppat-0020049-b027] Loetscher P, Seitz M, Clark-Lewis I, Baggiolini M, Moser B (1996). Activation of NK cells by CC chemokines. Chemotaxis, Ca^2+^ mobilization, and enzyme release. J Immunol.

[ppat-0020049-b028] Drake PM, Gunn MD, Charo IF, Tsou CL, Zhou Y (2001). Human placental cytotrophoblasts attract monocytes and CD56(bright) natural killer cells via the actions of monocyte inflammatory protein 1alpha. J Exp Med.

[ppat-0020049-b029] Johnson LL (1992). SCID mouse models of acute and relapsing chronic Toxoplasma gondii infections. Infect Immun.

[ppat-0020049-b030] Luangsay S, Kasper LH, Rachinel N, Minns LA, Mennechet FJ (2003). CCR5 mediates specific migration of Toxoplasma gondii-primed CD8 lymphocytes to inflammatory intestinal epithelial cells. Gastroenterology.

[ppat-0020049-b031] Aliberti J, Valenzuela JG, Carruthers VB, Hieny S, Andersen J (2003). Molecular mimicry of a CCR5 binding-domain in the microbial activation of dendritic cells. Nat Immunol.

[ppat-0020049-b032] Zhou Y, Kurihara T, Ryseck R-P, Yang Y, Ryan C (1998). Impaired macrophage function and enhanced T cell-dependent immune response in mice lacking CCR5, the mouse homologue of the major HIV-1 coreceptor. J Immunol.

[ppat-0020049-b033] Dawson TC, Beck MA, Kuziel WA, Henderson F, Maeda N (2000). Contrasting effects of CCR5 and CCR2 deficiency in the pulmonary inflammatory response to influenza A virus. Am J Pathol.

[ppat-0020049-b034] Sato N, Kuziel WA, Melby PC, Reddick RL, Kostecki V (1999). Defects in the generation of IFN-gamma are overcome to control infection with Leishmania donovani in CC chemokine receptor (CCR) 5-, macrophage inflammatory protein-1 alpha-, or CCR2-deficient mice. J Immunol.

[ppat-0020049-b035] Andres PG, Beck PL, Mizoguchi E, Mizoguchi A, Bhan AK (2000). Mice with a selective deletion of the CC chemokine receptors 5 or 2 are protected from dextran sodium sulfate-mediated colitis: Lack of CC chemokine receptor 5 Expression results in a NK1.1^+^ lymphocyte-associated Th2-type immune response in the intestine. J Immunol.

[ppat-0020049-b036] Deng H, Liu R, Ellmeier W, Choe S, Unutmaz D (1996). Identification of a major co-receptor for primary isolates of HIV-1. Nature.

[ppat-0020049-b037] Dragic T, Litwin V, Allaway GP, Martin SR, Huang Y (1996). HIV-1 entry into CD4^+^ cells is mediated by the chemokine receptor CC-CKR-5. Nature.

[ppat-0020049-b038] Dean M, Carrington M, Winkler C, Huttley GA, Smith MW (1996). Genetic restriction of HIV-1 infection and progression to AIDS by a deletion allele of the CKR5 structural gene. Science.

[ppat-0020049-b039] Samson M, Libert F, Doranz BJ, Rucker J, Liesnard C (1996). Resistance to HIV-1 infection in caucasion individuals bearing mutant alleles of the CCR-5 chemokine receptor gene. Nature.

[ppat-0020049-b040] Liu R, Paxton WA, Choe S, Ceradini D, Martin SR (1996). Homozygous defect in HIV-1 coreceptor accounts for resistance of some multiply-exposed individuals to HIV-1 infection. Cell.

[ppat-0020049-b041] Gomez-Reino JJ, Pablos JL, Carreira PE, Santiago B, Serrano L (1999). Association of rheumatoid arthritis with a functional chemokine receptor, CCR5. Arthritis Rheum.

[ppat-0020049-b042] Zapico I, Coto E, Rodriguez A, Alvarez C, Torre JC (2000). CCR5 (chemokine receptor-5) DNA-polymorphism influences the severity of rheumatoid arthritis. Genes Immun.

[ppat-0020049-b043] Garred P, Madsen HO, Petersen J, Marquart H, Hansen TM (1998). CC chemokine receptor 5 polymorphism in rheumatoid arthritis. J Rheumatol.

[ppat-0020049-b044] Rector A, Vermeire S, Thoelen I, Keyaerts E, Struyf F (2001). Analysis of the CC chemokine receptor 5 (CCR5) delta-32 polymorphism in inflammatory bowel disease. Hum Genet.

[ppat-0020049-b045] Bennetts BH, Teutsch SM, Buhler MM, Heard R.N, Stewart GJ (1997). The CCR5 deletion mutation fails to protect against multiple sclerosis. Hum Immunol.

[ppat-0020049-b046] Barcellos LF, Schito AM, Rimmler JB, Vittinghoff E, Shih A (2000). CC-chemokine receptor 5 polymorphism and age of onset in familial multiple sclerosis. Multiple Sclerosis Genetics Group. Immunogenetics.

[ppat-0020049-b047] Hokeness KL, Kuziel WA, Biron CA, Salazar-Mather TP (2005). Monocyte chemoattractant protein-1 and CCR2 interactions are required for IFN-alpha/beta-induced inflammatory responses and antiviral defense in liver. J Immunol.

[ppat-0020049-b048] Kuziel WA, Maeda N, Mak T (1998). CCR5. The gene knockout factsbook.

[ppat-0020049-b049] Burg JL, Grover CM, Pouletty P, Boothroyd JC (1989). Direct and sensitive detection of a pathogenic protozoan, *Toxoplasma gondii,* by polymerase chain reaction. J Clin Microbiol.

[ppat-0020049-b050] Kirists MJ, Mui E, Mcleod R (2000). Measurement of the efficacy of vaccines and antimicrobial therapy against infection with Toxoplasma gondii. Int J Parasit.

[ppat-0020049-b051] Watanabe H, Ohtsuka K, Kimura M, Ikarashi Y, Ohmori K (1992). Details of an isolation method for hepatic lymphocytes in mice. J Immunol Methods.

[ppat-0020049-b052] Khan IA, Moretto M, Wei XQ, Williams M, Schwartzman JD (2002). Treatment with soluble interleukin-15R alpha exacerbates intracellular parasitic infection by blocking the development of memory CD8^+^ T cell response. J Exp Med.

[ppat-0020049-b053] Means TK, Hayashi F, Smith KD, Aderem A, Luster AD (2003). The Toll-like receptor 5 stimulus bacterial flagellin induces maturation and chemokine production in human dendritic cells. J Immunol.

[ppat-0020049-b054] Arase H, Saito T, Phillips JH, Lanier LL (2001). Cutting edge: The mouse NK cell-associated antigen recognized by DX5 monoclonal antibody is CD49b (alpha 2 integrin, very late antigen-2). J Immunol.

